# Paclitaxel, vinorelbine and 5-fluorouracil in breast cancer patients pretreated with adjuvant anthracyclines

**DOI:** 10.1038/sj.bjc.6602335

**Published:** 2005-01-25

**Authors:** A Berruti, R Bitossi, G Gorzegno, A Bottini, D Generali, M Milani, D Katsaros, I A Rigault de la Longrais, R Bellino, M Donadio, M Ardine, O Bertetto, S Danese, M G Sarobba, A Farris, V Lorusso, L Dogliotti

**Affiliations:** 1 Oncologia Medica, Azienda Ospedaliera San Luigi, Regione Gonzole 10, 10043 Orbassano (TO), Italy; 2 Breast Unit, Azienda Ospedaliera Istituti Ospitalieri, largo Priori, 26100 Cremona, Italy; 3 Ginecologia Oncologica, Azienda Ospedaliera OIRM Sant'Anna, via Ventimiglia 3, 10126 Torino, Italy; 4 Oncologia Medica, Centro Oncologico Ematologico Subalpino, Azienda Ospedaliera San Giovanni Battista Molinette, corso Bramante 88, 10126 Torino, Italy; 5 Ginecologia Divisione A, Azienda Ospedaliera OIRM Sant'Anna, corso Spezia 60, 10126 Torino, Italy; 6 Oncologia Medica, Istituto Clinica Medica Universitaria, via San Pietro 8, 07100 Sassari, Italy; 7 Oncologia Medica, Istituto Oncologico, via Amendola 209, 70126 Bari, Italy

**Keywords:** breast cancer, metastatic disease, paclitaxel, vinorelbine, 5-fluorouracil

## Abstract

We investigated the activity and toxicity of a combination of vinorelbine (VNB), paclitaxel (PTX) and 5-fluorouracil (5-FU) continuous infusion administered as first-line chemotherapy in metastatic breast cancer patients pretreated with adjuvant anthracyclines. A total of 61 patients received a regimen consisting of VNB 25 mg m^−2^ on days 1 and 15, PTX 60 mg m^−2^ on days 1, 8 and 15 and continuous infusion of 5-FU at 200 mg m^−2^ every day. Cycles were repeated every 28 days. Disease response was evaluated by both RECIST and World Health Organization (WHO) criteria. Objective responses were recorded in 39 of 61 patients (64.0%) assessed by WHO and in 36 of 50 patients (72.0%) assessable by RECIST criteria. Complete remission occurred in 15 (24.6%) and 14 patients (28.0%), respectively. The median time to progression and overall survival of entire population was 10.6 and 27.3 months, respectively, and median duration of complete response was 14.8 months. The dose-limiting toxicity was myelosuppression (leucopenia grade 3/4 in 52.5% of patients). Grade 3/4 nonhaematologic toxicities included mucositis/diarrhoea in 13.1%, skin in 3.3% and cardiac in 1.6% of patients. Grade 2/3 neurotoxicity was observed in five patients (7.2%). The VNB, PTX and 5-FU continuous infusion combination regimen was active and manageable. Complete responses were frequent and durable.

Breast cancer is the most commonly diagnosed cancer and remains the first cause of cancer-related death in women in Italy (Negri *et al*, 2001). In the management of patients with metastatic disease, chemotherapy may offer effective palliation, and anthracyclines are widely recognised as the most active drugs (Esteva *et al*, 2001; Crown *et al*, 2002). The widespread use of these agents in the adjuvant setting, however, limits their employment in disease recurrence, thus demanding the testing of new effective regimens. In the past decade, vinorelbine and taxanes have been shown to generate a consistent level of activity in advanced breast cancer, yielding as single agents overall response rates between 30.0 and 50.0% along with acceptable toxicity profiles. Both drugs were demonstrated to be relatively non-crossresistant to anthracyclines (Esteva *et al*, 2001; Crown *et al*, 2002).

Vinorelbine blocks the formation of the mitotic spindle apparatus at the metaphase by inhibiting microtubule assembly (Fellous *et al*, 1989). Conversely, paclitaxel promotes and stabilises polymerised tubulin into nonfunctional microtubule bundles, thus blocking the cells in the G2/M phase of the cell cycle (Manfredi and Horwitz, 1984). The combination of vinorelbine and paclitaxel is thought to create a total microtubule poison that offers theoretical advantages deriving from the potential synergy between the two drugs when given together. Preclinical data have shown that paclitaxel and vinorelbine were synergistic *in vitro* (Photiou *et al*, 1997; Budman *et al*, 2000), while *in vivo* their combination produced a significantly greater proportion of cell kill of transplanted p388 murine leukaemia cells than either agent alone (Knick *et al*, 1995). The combination of paclitaxel and vinorelbine has been evaluated in a number of phase I and II trials. As a whole, the results have been encouraging, with an overall response rate between 47.4 and 67.0% (Martin *et al*, 1998; Tortoriello *et al*, 1998; Culine *et al*, 1999; Ellis *et al*, 1999; Romero Acuna *et al*, 1999; Vici *et al*, 2000; Ibrahim *et al*, 2001; Ballestrero *et al*, 2003).

Fluoropyrimidines, and 5-fluorouracil (5-FU) in particular, have been employed for over 40 years to treat numerous solid tumours including breast cancer (Venturini, 2002). Fluorouracil is an S-phase-specific agent with a short serum half-life of 10–20 min, making it active only against a small proportion of tumour cells in the S phase when administered via short intravenous bolus injection. When it is given over an extended period of time, the resulting greater number of actively dividing cells exposed to the drug warrants the administration of 5-FU as a continuous infusion (Cameron *et al*, 1994; Regazzoni *et al*, 1996). In addition, due to the modified plasma concentration profile, large cumulative fluorouracil doses administered via continuous infusion are better tolerated than bolus injection (Cameron *et al*, 1994; Regazzoni *et al*, 1996). The moderate myelotoxicity of infusional 5-FU allows its combination with other myelotoxic agents. The theoretical advantages of the association of infusional 5-FU with paclitaxel and vinorelbine include (1) 5-FU has a different mechanism of cytotoxicity, (2) its proposed mechanism of resistance is different and (3) it possesses relatively nonoverlapping toxicity. The therapeutic activity of fluorouracil combined with vinorelbine or paclitaxel in metastatic breast cancer has been widely tested, and high response rates (up to 70.0% as a first-line approach) have been reported (Vredenburgh *et al*, 1998). To our knowledge, the association of protracted infusion of 5-FU with both vinorelbine and paclitaxel has never been tested. In a phase II trial we conducted, biweekly vinorelbine associated with protracted 5-FU infusion was found to be extremely active as a second- or third-line approach in advanced breast cancer patients previously treated with anthracyclines (Berruti *et al*, 2000). In view of these encouraging results, we tested this combination in association with paclitaxel as first-line treatment in advanced breast cancer patients pretreated in adjuvant setting with anthracycline-containing regimens. The primary aim of the study was to evaluate the activity of the combination regimen; the secondary aim was to assess toxicity, time to progression (TTP) and overall survival (OS).

## PATIENTS AND METHODS

### Patients

Women, 18 years of age or older, were eligible for the study if they had histologically confirmed advanced breast cancer and disease assessable according to the World Health Organization (WHO) criteria of disease response (22). Other eligibility criteria included good performance status (WHO grade 0–1), adequate bone marrow reserve (WBC count ⩾3.5 × 10^9^ l^−1^, platelets ⩾100 × 10^9^ l^−1^), adequate hepatic and renal function (hepatic enzymes and bilirubin <2 × upper limit of normal, serum creatinine within normality) and an estimated life expectancy of at least 12 weeks. Patients were excluded from the study who presented with nonmalignant systemic disease or conditions that precluded them from receiving study therapy (e.g. active infection, any clinically significant arrhythmia, congestive heart failure or pregnancy) or with CNS metastases or second primary malignancies (except *in situ* carcinoma of the cervix or adequately treated basal cell carcinoma of the skin), or who used any investigational agent 1 month before enrolment. All patients had to have received adjuvant chemotherapy with an anthracycline-containing regimen. Prior systemic chemotherapy for advanced disease and prior exposure to either vinorelbine or taxanes were not allowed, but one line of endocrine therapy in the metastatic setting was permitted. The study was approved by the local ethics committee. Written informed consent was obtained from all patients before starting treatment.

### Treatment schedule

Treatment consisted of: vinorelbine (Navelbine; Pierre Fabre Pharma, Milan, Italy) 25 mg m^−2^ on days 1 and 15 every 28 days given as a 10-min infusion in 100 ml saline solution; paclitaxel (Taxol; Bristol Myers Squibb, Rome, Italy) 60 mg m^−2^ on days 1, 8 and 15 every 28 days given as a 3-h infusion in 500 ml normal saline solution; 5-FU given at a daily dose of 200 mg m^−2^ as a protracted continuous infusion using an elastomeric pump. All patients had a central venous access. All drugs were administered on an outpatient basis. Since preclinical data suggested that the sequence of administration of paclitaxel and vinorelbine was predictive for treatment efficacy (Photiou *et al*, 1997; Budman *et al*, 2000), vinorelbine infusion preceded paclitaxel infusion. Dose modifications were performed as follows: in case of myelosuppression, if the WBC count was ⩽2500 *μ*l^−1^ and/or the platelet count was less than 100 000 *μ*l^−1^, then 5-FU was continued but both paclitaxel and vinorelbine at that day were omitted. If the blood count had recovered after 1 week, full-dose vinorelbine and paclitaxel were then administered. If the blood count had not recovered, then both vinorelbine and paclitaxel were further omitted, and the drug dose was subsequently reduced by 25%. In the event of hand and foot syndrome, for mild to moderate palmoplantar erythema (dryness and erythema with pain), patients continued 5-FU; for severe palmoplantar erythema with blistering and desquamation, 5-FU was interrupted until the erythema had returned to grade 1 or less, and afterwards 5-FU was restarted at full doses. For WHO grade 1 or 2 diarrhoea, antidiarrhoeal agents were prescribed; for persistent diarrhoea, 5-FU, but not vinorelbine or paclitaxel, was discontinued for 1 week. In case of resolution, 5-FU was restarted at full doses; if symptoms persisted, a 25% dose reduction was performed. For grade 3 or 4 diarrhoea, 5-FU was withdrawn until the diarrhoea had returned to grade 1 or less, and afterwards 5-FU was restarted with a 50% reduction in dosage. In patients with grade 2 mucositis, infusional 5-FU was stopped for 1 week, then restarted at full doses; if symptoms recurred, a 25% dose reduction was performed. For grade 3 or 4 mucositis, 5-FU was withdrawn until complete resolution (grade 0) and restarted with a 25% reduction in dosage. Vinorelbine and paclitaxel doses were delayed for 1 week in case of grade 2 neurotoxicity; in the event of grade 3 neurotoxicity, vinorelbine was reduced by 50% in subsequent cycles, whereas paclitaxel was withdrawn.

Relative dose intensity was defined as the actual weekly doses of vinorelbine, paclitaxel and 5-FU at the end of treatment divided by the planned weekly dose. Supportive care could include blood transfusion and administration of analgesics, antiemetics and growth factors as appropriate. The prophylactic use of G-CSF to maintain dose intensity was not permitted.

### Assessment of response and toxicity

Pretreatment evaluation included medical history and physical examination, complete blood cell count, serum chemistries, liver function tests, ECG, echocardiography, tumour marker evaluation (CA 15-3) and staging studies appropriate to define the extent of metastatic disease, including chest X-ray, abdominal ultrasonography, thoracic and/or abdominal computed tomography scanning and bone scanning. Clinical monitoring was performed once weekly; complete blood cell counts, serum electrolytes and liver function tests were performed every 2 weeks. Toxicity was evaluated according to the WHO criteria (Miller *et al*, 1981).

Antitumour activity was evaluated every 3 months on all measurable lesions, and all patients were scheduled for at least a 2-month treatment in order to be eligible for assessment of tumour response. The response obtained needed confirmation after at least 1 month. In patients with tumour response or stable disease, the treatment was planned to be continued for up to 6 months; thereafter, maintenance or no endocrine therapy (in patients with ER+ tumours) was based on the clinician's choice. After the completion of chemotherapy, the patients were monitored every 3 months.

Tumour response was classified according to either the WHO criteria (Miller *et al*, 1981) or the RECIST criteria (Therasse *et al*, 2000), as detailed elsewhere. All deaths and treatment discontinuations (for toxicity or patient refusal) were considered as treatment failures. Time to progression was calculated from the beginning of cytotoxic chemotherapy until the date of objective evidence of progressive disease. Survival was dated from the first day of treatment until death or was censored on the date of the last follow-up appointment.

### Statistical analysis

The primary study end point was the assessment of the response rate (intent-to-treat analysis). According to the optimal two-stage phase II study design of Simon (1989), the sample size was assessed in order to refuse response rates ⩽40.0% (p0) and to provide a statistical power of 80.0% in assessing the activity of the regimen as a 60.0% response rate.

A maximum of 46 consecutive cases were required with 16 cases in the first stage and 30 in the second stage. If seven responses or fewer were observed in the first stage, then the trial was stopped with the conclusion that the success rate was less than 40.0%. If greater than seven responses were observed, then the trial was continued to the second stage to recruit a further 30 consecutive patients. Since both WHO and RECIST criteria were employed to assess disease response, the trial was planned to end when at least 46 cases assessable with the two response criteria had been consecutively enrolled. Response duration and survival were assessed using Kaplan–Meier survival curves. A two-sided significance of the 5.0% level was applied to all tests. All statistical analyses were performed using the Statistica for Windows software program.

## RESULTS

From January 2000 to April 2002, 61 consecutive patients were enrolled in the study. Of these, all were assessable according to WHO criteria and 50 patients were also assessable according to RECIST response criteria. The demographic data, metastatic tumour sites and prior therapies are listed in Table 1. In all, 34 (55.8%) patients had one metastatic site and 27 (44.2%) had multiple metastases involving two or more organ systems. Predominant visceral sites were found in 43 patients (70.5%), whereas predominant bone and soft tissue sites were found in 11 (14.7%) and seven (11.5%), respectively.

### Treatment activity

The best responses recorded for each patient are listed in Table 2. According to the WHO criteria, an objective regression was recorded in 39 women (64.0% (95% confidence interval (CI) 51.7–76.3%)), of which 15 (24.6%) attained a complete clinical response. The distribution of responses according to the disease site is listed in Table 3. Overall response in patients with only one disease site (21 out of 35, 60.0%) was similar to that of patients bearing multiple sites of disease (17 out of 26, 65.4%). At the last follow-up appointment (30 November 2003), 47 patients (77.0%) showed disease progression and 33 (54.1%) had died. Median TTP and OS of the entire group was 10.6 and 27.3 months, respectively. Median duration of disease response was 10.2 months (14.8 months in complete responders). Stratifying patients according to the disease-free interval (DFI), disease response occurred in nine out of 18 patients (50.0%) with DFI less than 2 years while it obtained in 30 out of 43 patients (69.8%) with DFI more than 2 years (*P*=0.14). The corresponding median TTP was 6.7 and 13.8 months in the two groups, respectively (*P*=0.003).

The treatment activity evaluated by RECIST criteria was 36 out of 50 assessable cases with a disease response (72.0% (95% CI 59.3–84.7%)) and 14 (28.0%) with a complete clinical response. Median TTP and OS in this patient subset was 10.0 and 25.5 months, respectively. Median duration of disease response was 9.4 months (17.8 months in complete responders).

### Toxicity

A total of 309 cycles of therapy were administered (median six cycles; range 1–6). Associated side effects are reported in Table 4. Haematological toxicity was the most frequent severe toxicity. Grade 3–4 leucopenia was observed in 52.5% of patients; grade 3–4 Hb toxicity was recorded in 6.6% of patients. Gastrointestinal toxicities included grade 3 nausea and vomiting (6.6%), grade 3 mucositis (11.5%) and grade 3 hepatic toxicity (3.3%). Two patients experienced serious hypersensitivity reactions after the second paclitaxel infusion. Paclitaxel was therefore interrupted in these patients, while vinorelbine and 5-FU were maintained. A total of 18 patients (29.5%) developed neurologic toxicity, with grades 2 and 3 recorded in five cases (8.2%), and dose-limiting toxicity in three. It was noteworthy that more than 70.0% of patients did not develop alopecia.

In a total of 24 (39.3%) patients, 64 courses (20.7%) were delayed 1 week, and eight courses (2.6%) (six patients (9.8%)) were delayed 2 weeks due to haematological toxicity. Paclitaxel was reduced or omitted in 39 patients (63.9%) (107 courses (34.6%)), while vinorelbine and 5-FU were reduced or omitted in 29 (47.5%) (64 courses (20.7%)) and in 37 (60.6%) (99 courses (32.0%)), respectively. Leucopenia was the most frequent cause of dose reduction/omission of the three drugs. Paclitaxel was reduced in three patients (six cycles) due to neurotoxicity; 5-FU was reduced/omitted in four (12 courses) due to gastrointestinal toxicity (diarrhoea, mucositis), in one patient (two courses) due to skin toxicity and in seven (12 courses) due to central venous access problems (delayed positioning in five patients, catheter infection or thrombosis in two). A total of 42 patients (68.8%) ended the treatment plan (six cycles), four (6.6%) received five cycles, two (3.3%) received four cycles, seven (11.5%) received three cycles, four (6.6%) received two cycles and two (3.3%) received one cycle. The reasons for early stopping treatment were disease progression (six patients), leucopenia (six patients), mucositis (one patient), hepatic toxicity (one patient), cardiac toxicity (one patient), patient refusal (three patients) and sudden death (one patient).

Dose intensity was calculated for each patient and for each drug. The median dose intensity of vinorelbine was 10.7 mg week^−1^ (85.7% of planned dose), the median dose intensity of paclitaxel was 37.2 mg week^−1^ (82.7%) and the median dose intensity of 5-FU was 1187.9 mg week^−1^ (84.8%).

## DISCUSSION

The rationale for administering vinorelbine in association with paclitaxel in anthracycline-pretreated breast cancer patients centres mainly on the documented activity of the two drugs in anthracycline refractory patients (Crown *et al*, 2002) and on the synergism of the cytotoxic effects of the two drugs in preclinical models (Photiou *et al*, 1997; Budman *et al*, 2000). The addition of infusional 5-FU might further increase the activity of this combination.

In a previous study our cooperative group conducted, biweekly vinorelbine associated with continuous 5-FU infusion was found to be well tolerated and highly active in a subgroup of heavily pretreated patients (Berruti *et al*, 2000). In the present study, paclitaxel was added to this regimen. Since weekly paclitaxel infusion has been reported to be more active than 3-weekly administration (Seidman *et al*, 1998), paclitaxel was administered on a sustained weekly schedule.

Our results suggest that vinorelbine, paclitaxel and 5-FU have a high antitumoral activity in anthracycline-pretreated patients, as indicated by the overall response rate, median TTP and OS.

This trial was designed when the new RECIST criteria for response assessment had just been proposed to replace the WHO criteria. So we thought it would be interesting to look at both criteria to assess disease response. Since the RECIST criteria are more restrictive than the WHO criteria (e.g. patients with bone metastases alone are admitted on WHO criteria but not on RECIST criteria), the number of enrolled cases eligible for WHO response exceeded by 20% the number of enrolled cases eligible for RECIST response. The overall response rate in the 50 patients eligible for assessment by either WHO or RECIST criteria was quite similar, but when all consecutively assessable patients were considered, the disease response assessed by the RECIST criteria was slightly higher than that assessed by the WHO criteria. The difference is mainly due to the inclusion of bone-only metastases in patients eligible on WHO criteria, which are notoriously less responsive than visceral metastases (Coleman and Rubens, 1987). The 64.0% response rate recorded in this trial using the WHO criteria (72.0% on the RECIST criteria) is noteworthy when we consider that all patients had previously received anthracyclines. The therapeutic activity of the regimen was not influenced by adverse prognostic factors such as predominant visceral disease or multiple metastatic sites. Conversely, patients with short DFI had a tendency to low disease response and shorter TTP as opposed to their counterparts, thus confirming previous observations showing that DFI is an important predictor of poor outcome in metastatic breast cancer patients treated with first-line therapy (Kramer *et al*, 2000). The proportion of patients attaining a clinical complete response (24.6 and 28.0% by WHO and RECIST criteria, respectively) is quite impressive. Complete responses were observed in all metastatic sites, and particularly in the liver. Complete clinical response was durable (20 months on average), a noteworthy finding since liver metastases are associated with poor prognosis (Atalay *et al*, 2003).

As concerns treatment tolerability, leucopenia was the most frequent and dose-limiting side effect associated with the regimen. WHO grade 3 or 4 WBC toxicity occurred in 52.5% of patients, although it was never complicated by sepsis requiring hospitalisation. Since the prophylactic use of G-CSF was not permitted, leucopenia caused frequent reduction/omission, mainly of paclitaxel and/or vinorelbine. The frequency of bone marrow depression observed here did not differ from that reported in other phase II studies testing the association of paclitaxel and vinorelbine (Martin *et al*, 1998; Tortoriello *et al*, 1998; Culine *et al*, 1999; Ellis *et al*, 1999; Romero Acuna *et al*, 1999; Vici *et al*, 2000; Ibrahim *et al*, 2001; Ballestrero *et al*, 2003). This suggests that 5-FU may have influenced this side effect only marginally. 5-Fluorouracil was less frequently reduced due to haematologic toxicity, but drug doses were adjusted due to mucositis, central catheter complications and, less frequently, because of hand and foot syndrome. Neurotoxicity is an expected side effect of both paclitaxel and vinorelbine; this side effect may potentially be increased by the association of the two drugs. A total of 18 patients developed neurotoxicity, but most showed only minor neurologic impairment. Grade 2–3 neurotoxicity was observed in five cases, and only three required transient paclitaxel withdrawal. Other reported treatment-related toxicities were clinically unremarkable.

In conclusion, the association of paclitaxel, vinorelbine and infusional 5-FU shows favourable activity and toxicity profiles and it may provide a suitable therapeutic option in advanced breast cancer patients pretreated with anthracyclines. Although we were unable to consistently deliver the planned full drug dose in all patients, the activity of this combination was significant and the responses were durable. Whether 5-FU could offer additional benefit to the association of paclitaxel and vinorelbine needs to be addressed in a randomised clinical trial.

## Figures and Tables

**Table 1:** Patient characteristics

Characteristics	Patients (*N*=61)
*Age (years)*	
Median (range)	54.3 (30.6–70.7)
Postmenopause	54 (88.5%)
Premenopause	7 (11.5%)
*Performance status* a	
0	40 (65.6%)
1	14 (22.9%)
2	5 (8.2%)
3	2 (3.3%)
*Oestrogen receptor status*	
Positive	30 (49.2%)
Negative	26 (42.6%)
Unknown	5 (8.2%)
Disease-free interval (months)	43.5 (9–265.7)
*Previous treatments*	
Surgery	60 (98.4%)
Radiation therapy	26 (42.6%)
Adjuvant anthracycline chemotherapy	61 (100.0%)
*Previous endocrine therapy*	
Adjuvant	27 (44.3%)
Advanced disease	11 (18.0%)
*Disease sites*	
Skin/lymph nodes	20 (32.8%)
Bone	27 (44.3%)
Lung	29 (47.5%)
Liver	22 (36.1%)
Other	3 (4.9%)
*Number of sites of disease*	
1	34 (55.8%)
2	16 (26.2%)
3	10 (16.4%)
4	1 (1.6%)

a ECOG scale.

**Table 2:** Treatment activity

	**WHO criteria**	**RECIST criteria**
	**Patients (*N*=61)**	**Patients (*N*=50)**
Not evaluable	5 (8.2%)	4 (8.0%)
*Reasons*		
Treatment refusal	2 (3.3%)	1 (2.0%)
Death	1 (1.6%)	1 (2.0%)
Toxicity	2 (3.3%)	2 (4.0%)
		
Progressive disease	6 (9.8%)	4 (8.0%)
Stable disease	11 (18.0%)	6 (12.0%)
Partial response	24 (39.4%)	22 (44.0%)
Complete response	15 (24.6%)	14 (28.0%)
		
Overall response	39 (64.0%)	36 (72.0%)
(95% Confidence interval)	(51.7–76.3%)	(59.3–84.7%)

**Table 3:** Disease response according to sites of disease (WHO)

**Disease site**	**CR**	**PR**	**SD**	**PD**
Skin/lymph (total no., *n*=19)	8 (42.1%)	6 (31.6%)	2 (10.5%)	3 (15.8%)
				
Bone (total no., *n*=27)	2 (7.4%)	9 (33.3%)	11 (40.8%)	5 (18.5%)
				
Lung (total no., *n*=29)	7 (24.1%)	10 (34.5%)	8 (27.6%)	4 (13.7%)
				
Liver (total no., *n*=22)	9 (40.9%)	4 (18.2%)	3 (13.6%)	6 (27.2%)

**Table 4:** Toxicity

**Grade 0**	**1**	**2**	**3**	**4**
Leucopenia 7 (11.5%)	6 (9.8%)	16 (26.2%)	22 (36.1%)	10 (16.4%)
Anaemia 23 (37.7%)	25 (41.0%)	9 (14.7%)	4 (6.6%)	—
Thrombocytopenia 57 (93.5%) 1 (1.6%)	1 (1.6%)	1 (1.6%)	1 (1.6%)	
Nausea/vomiting 34 (55.7%) 17 (27.9%)	6 (9.8%)	4 (6.6%)	—	
Diarrhoea 48 (78.7%)	9 (14.7%)	3 (4.9%)	1 (1.6%)	—
Mucositis 40 (65.6%)	8 (13.1%)	6 (9.8%)	7 (11.5%)	—
Hepatic 59 (96.7%)	—	—	2 (3.3%)	—
Myalgias 55 (90.2%)	4 (6.6%)	2 (3.2%)	—	—
Fever 55 (90.2%)	3 (4.9%)	3 (4.9%)	—	—
Neurological 43 (70.5%)	13 (21.3%)	4 (6.6%)	1 (1.6%)	—
Cardiac 56 (91.8%)	4 (6.6%)	—	1 (1.6%)	—
Skin 48 (78.6%)	7 (11.5%)	4 (6.6%)	2 (3.3%)	—
Alopecia 44 (72.1%)	1 (1.6%)	2 (3.3%)	14 (23.0%)	—

No patients had lung or bladder toxicity.
